# Left ventricular strain and peak systolic velocity: responses to controlled changes in load and contractility, explored in a porcine model

**DOI:** 10.1186/1476-7120-10-22

**Published:** 2012-05-28

**Authors:** Roman A’roch, Ulf Gustafsson, Göran Johansson, Jan Poelaert, Michael Haney

**Affiliations:** 1 Department of Surgical and Perioperative Sciences, Anaesthesiology and Intensive Care Medicine, University Hospital of Umeå, Umeå, Sweden; 2 Department of Clinical Physiology, Heart Centre, University Hospital of Umeå, Umeå, Sweden; 3 Department of Surgical and Perioperative Sciences, Anaesthesiology and Intensive Care Medicine, University Hospital of Umeå, Umeå, Sweden; 4 Department of Anaesthesiology and Perioperative Medicine, University Hospital of Brussels, Brussels, Belgium; 5 Department of Surgical and Perioperative Sciences, Anaesthesiology and Intensive Care Medicine, University Hospital of Umeå, Umeå, Sweden

**Keywords:** Tissue velocities echocardiography, Ventricular function, Load

## Abstract

**Background:**

Tissue velocity echocardiography is increasingly used to evaluate global and regional cardiac function. Previous studies have suggested that the quantitative measurements obtained during ejection are reliable indices of contractility, though their load-sensitivity has been studied in different settings, but still remains a matter of controversy. We sought to characterize the effects of acute load change (both preload and afterload) and change in inotropic state on peak systolic velocity and strain as a measure of LV contractility.

**Methods:**

Thirteen anesthetized juvenile pigs were studied, using direct measurement of left ventricular pressure and volume and transthoracic echocardiography. Transient inflation of a vena cava balloon catheter produced controlled load alterations. At least eight consecutive beats in the sequence were analyzed with tissue velocity echocardiography during the load alteration and analyzed for change in peak systolic velocities and strain during same contractile status with a controlled load alteration. Two pharmacological inotropic interventions were also included to generate several myocardial contractile conditions in each animal.

**Results:**

Peak systolic velocities reflected the drug-induced changes in contractility in both radial and longitudinal axis. During the acute load change, the peak systolic velocities remain stable when derived from signal in the longitudinal axis and from the radial axis. The peak systolic velocity parameter demonstrated no strong relation to either load or inotropic intervention, that is, it remained unchanged when load was systematically and progressively varied (peak systolic velocity, longitudinal axis, control group beat 1-5.72 ± 1.36 with beat 8–6.49 ± 1.28 cm/sec, 95% confidence interval), with the single exception of the negative inotropic intervention group where peak systolic velocity decreased a small amount during load reduction (beat 1–3.98 ± 0.92 with beat 8–2.72 ± 0.89 cm/sec). Systolic strain, however, showed a clear degree of load-dependence.

**Conclusions:**

Peak systolic velocity appears to be load-independent as tested by beat-to-beat load reduction, while peak systolic strain appears to be load-dependent in this model. Peak systolic velocity, in a controlled experimental model where successive beats during load alteration are assessed, has a strong relation to contractility. Peak systolic velocity, but not peak strain rate, is largely independent of load, in this model. More study is needed to confirm this finding in the clinical setting.

## Introduction

Since its introduction tissue velocity echocardiography (TVE) has opened new possibilities for non-invasive quantification of myocardial function [1]. Tissue Doppler echocardiography is an established cardiac diagnostic method for measurement and characterization of both the systolic and diastolic cardiac dysfunction [2-5]. Myocardial velocity is the most widely used and validated parameter from this method [6-8]. Through further derivation and integration of tissue velocities, additional parameters like displacement, strain rate and strain can be calculated. Strain is a dimensionless quantity, which is the product of stress. It represents the fractional change (or percentage) from the original or unstressed dimension. Positive strain indicates expansion or lengthening and negative strain compression or shortening.

An ideal index of myocardial contractility should be independent of loading conditions (preload and afterload), heart size and mass, and sensitive to changes in inotropy [9]. In this study, we have focused only on the issue of systolic velocities and load, since the load-dependence of diastolic tissue velocities has been well demonstrated [10,11].

The issue of load-dependency and tissue velocities during systole has been raised earlier, and clinical findings have been published that support the idea that load reduction can lead to decrase in systolic velocities [12] or alternatively increase in systolic velocity [13-15] , and in these cases, the authors have suggested mechanism other than load change (decrease) that lead to higher tissue velocities. Some preliminary findings for controlled load reduction in an experimental setting have shown that systolic velocities may decrease in conjunction with load decrease [16]. In the pediatric population with congenital heart disease, acute load reduction with inferior vena cava occlusion caused significant decrease in peak systolic velocities in the right ventricle [17].

We hypothesized that TVE systolic measures of the left ventricular function, peak systolic velocity (PSV) and systolic strain, would change in relation to loading changes. We further hypothesized that PSV and strain would behave in the same fashion during pharmacologically altered contractile status. We aimed to test these hypotheses in an experimental large animal model, which included exposure to positive and negative inotropic drugs and carefully controlled transient load changes. We also aimed to compare tissue velocities and strain measured from different directions with respect to myocardial axis.

## Methods

After approval from the Umeå Regional Animal Experimental Ethics Committee, and in conformation with the Guide for the Care and Use of Laboratory Animals (US National Academy of Sciences, 1996, USA), 13 juvenile Yorkshire/Hampshire pigs with a mean weight of 36.7 kg (SD = 4.1) were anaesthetized and instrumented, using methods that have been well described previously [18].

### Preparation

The animals were premedicated with ketamine 10 mg·kg^-1^, xylazine 2.2 mg·kg^-1^, and atropine 50 μg·kg^-1^. Anaesthesia was induced with pentobarbital 12 mg·kg^-1^ i.v. and maintained by a continuous infusion of pentobarbital 5 mg·kg^-1^·h^-1^, midazolam 0.3 mg·kg^-1^·h^-1^ and fentanyl 20 μg·kg^-1^·h^-1^. After tracheotomy, animals were intubated and ventilated in volume-controlled mode (Evita4, Dräger, Germany) to achieve normoxia and normocapnea (Artema, Artema Medical, Stockholm, Sweden) with a mixture of oxygen and room air. During the entire experiment IV fluids were administered: Ringer’s Acetate 15 ml·kg^-1^·h^-1^. Arterial and venous catheters were placed through cut downs in the cervical region to access the jugular and carotid vessel systems. Arterial and venous catheters, including a 7 F Swan-Ganz catheter (Optimetrix, Abbott, Illinois, USA) were advanced to appropriate position. A combined pressure-conductance catheter, with 12 electrodes and 8 mm spacing (CA-71083-PN, CD Leycom, Zoetermeer, Holland), was placed in the long axis with the pigtail tip in the apex of LV with the help of fluoroscopy. A 7.5 F balloon occlusion catheter (Vascular Technologies, Solna, Sweden) was placed in the inferior vena cava in order to facilitate a controlled transient restriction of venous return (VCBO maneuver) leading to a beat-by-beat progressive reduction in preload and afterload over a restricted range of normal operating pressures and volumes. Selected sequences from these load reduction periods were then further analyzed.

### Measurements

The method of left ventricular volume measurement with dual field conductance volume measurements is well described before elsewhere [19]. The conductance catheter was connected to a signal conditioning-amplifier set to dual-field mode (Leycom Sigma 5DF, CD Leycom, Zoetermeer, The Netherlands). Parallel conductance and flow reference ratio [20] were determined for LV volume calibration. Left ventricular pressure (Sentron, Roden, The Netherlands) and conductance data were recorded with a frequency of 250 Hz (PC Conduct, Cardiodynamics, Zoetermeer, The Netherlands). All circulatory parameters were recorded digitally and analyzed (Acqknowledge, Biopac Systems, Santa Barbara, California). Pressure-volume data analysis was performed with custom-made non-commercial software. Cardiac performance was assessed by heart rate, stroke volume, end-diastolic volume, end-systolic volume, cardiac output, and stroke work. Systolic load-dependent LV parameters include ejection fraction (EF), end-systolic pressure(P_es)_, maximal rate of pressure change (dP/dt_max_), and load-independent LV function parameters as the linear slope of the end-systolic PV relationship, defined as end-systolic elastance (E_es_) [21] and preload recruitable stroke work (PRSW) [22]. Diastolic load-dependent LV function was assessed by the LV end-diastolic pressure (P_ed_), isovolumic relaxation time constant (tau), minimal rate of LV pressure change (dP/dt_min_) and the ratio for end-systolic elastance/arterial elastance (Ees /Ea) [23].

Echocardiographic recordings, long axis and apical four chamber, were recorded with a frame rate of 100–140 frames per second using an ultrasound system (Vivid 7, GE Healthcare, Horten, Norway). Digitally stored data were analyzed offline using commercial software (EchoPac 6, GE Healthcare, Horten, Norway). Peak systolic velocities and strain was estimated by measuring the spatial velocity gradient over a computation area of 6x6 mm or 6x12 mm. The region of interest (ROI) was continuously positioned within the LV inferior wall when measuring radial measurements and within basal part of septum for longitudinal measurement. The strain length was chosen as 6 mm for posterior wall and 12 mm for septum [24]. The tissue tracking was done manually, frame-by-frame, to keep the ROI in the same position during the controlled transient restriction of venous return (VCBO maneuver) which included at least 8 consecutive beats. Baseline registrations were collected before each VCBO sequence.

### Protocol

Each measurement sequence was recorded during a period of apnea with 0 cm H_2_O airway pressure. The inferior vena cava balloon was inflated, and progressive beat-to-beat decreases in left ventricular pressure and volume were recorded. Beats selected for analysis from the balloon inflation period were those where there was a progressive beat-by-beat decrease in both LV end-diastolic and end-systolic volume and pressure at the beginning and end of the sequence. At least 8 consecutive beats, from the point where load reduction began to be noted were used for further analysis.

Concerning measurement sequences after inotropic interventions, a measurement was collected at baseline before the inotropic intervention was started, and then during adrenaline infusion with target pulse rate raise of approximately 20% from the baseline. After a second rest period, 30 minutes after discontinuation of adrenaline infusion, a negative inotropic intervention was administered, which consisted of an intravenous injection of metoprolol 40 mg and then verapamil 15 mg (all over a within a few short minutes), with a brief infusion of phenylephrine to counterbalance the immediate vasodilatatory effects of verapamil, which was quickly weaned off as soon as it was not needed.

### Statistics

Data are expressed as mean ± SEM or ± 95% confidence intervals. The effects of load alterations were analyzed using repeated measurements analysis of variance (repeated measures ANOVA). Comparison of the first beat (baseline) with the last (eighth) beat in an unloading sequence was performed using the paired Student t-test. A p value <0.05 was considered to be statistically significant.

## Results

**Figure 1:**
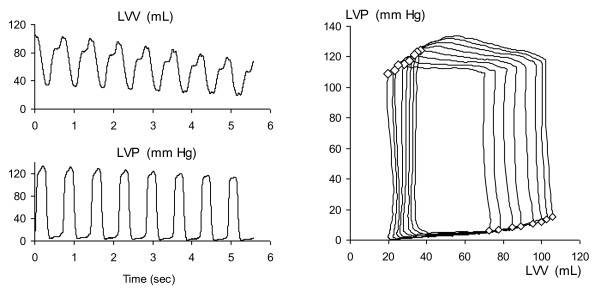
**A representative pressure-volume measurement sequence during controlled load reduction by transient vena cava balloon occlusion is shown.** This demonstrates that there are progressive beat-by-beat reductions in both pre- and post-systolic volumes and pressures, indicating systematic and progressive reduction in preload and afterload. The volumes and pressures for this measurement sequence are within the normal operating ranges for the heart and circulation.

**Figure 2:**
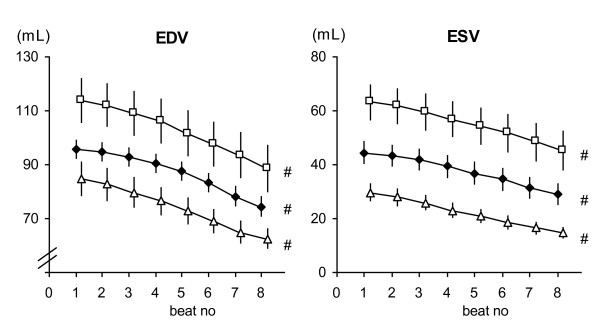
**End-diastolic and end-systolic volumes for the load alteration sequences are shown here, grouped by beat in the sequence.** All the first beats are grouped together, all the second beats, etc. The change in load, as demonstrated by volumes for the sequences collected during experimental alteration in inotropic status, is clearly shown. These load ranges correspond to the grouped tissue velocity and strain measures in later figures. Data are presented as mean ± SEM, n = 13. Filled diamond = Control; Open triangle = Adrenaline; Open square = Beta-blockade. EDV = end diastolic volume; ESV = end systolic volume. # p < 0.05 with repeated measures ANOVA.

The anaesthetized pigs all demonstrated central circulatory parameters consistent with health during rest before the start of the protocol. All 13 animals completed the protocol, with simultaneous tissue velocities echocardiography measurements together with LV pressure volume results for each sequence. Load changes were achieved by the VCBO, where the first eight beats in the sequences (Figure 1) are analyzed and a clear load alteration (reduction in end-diastolic volume, as well as reduction in stroke work) is shown, and this was consistent with all 3 inotropic conditions and in both projections (radial/short axis and longitudinal/long axis) (Figure 2).

Hemodynamic results show that the animals had their VCBO sequences with start and end ventricular pressures and volumes not in extreme ranges (Figures 1 and 2). Inotropic interventions, both positive and negative, achieved significant changes in contractile status as exemplified by change in PRSW from baseline 74.1 ± 10.2 mmHg to 104.0 ± 14.8 mmHg in positive inotropic status and to 51.3 ± 12.9 mmHg in negative inotropic status (Table 1).

**Table 1:** LVPVR parameters during control, adrenaline and beta-blockade

		**Control**	**Adrenalin**	**Beta blockade**
*Apnea measurement*				
HR	(bpm)	122 ± 14	122 ± 14 #	100 ±
SV	(mL)	55.2 ± 5.5	66.5 ± 12.6#	48.1 ± 8.3 #
CO	(L/min)	6.7 ± 0.9	9.6 ± 1.9 #	4.8 ± 0.9 #
V es	(mL)	40.9 ± 4.8	36.1 ± 8.0	68.3 ± 14.4 #
V ed	(mL)	92.5 ± 7.0	97.7 ± 15.8	113.9 ± 18.6 #
P es	(mm Hg)	121.3 ± 5.3	121.0 ± 9.6	105.0 ± 9.6 #
P ed	(mm Hg)	14.6 ± 3.3	14.1 ± 3.9	18.7 ± 3.2
dPdt max	(mm Hg/s)	2745 ± 252	5532 ± 752 ±	1557 ± 318 #
EF	(%)	59.7 ± 3.6	68.1 ± 7.4 #	43.0 ± 5.6 #
SW	(mm Hg·mL)	6227 ± 697	8215 ± 1471 #	4306 ± 926 #
Tau	(ms)	34.3 ± 3.0	23.3 ± 1.8 #	46.4 ± 12.3
PHT	(ms)	21.1 ± 1.5	14.6 ± 1.3 #	30.9 ± 7.0 #
dPdt min	(mm Hg·mL)	-2919 ± 175	-3587 ± 401 #	-1986 ± 289 #
PWR max	(mm Hg·mL/s)	44105 ± 5647	68020 ± 15038 #	32687 ± 5784 #
PWR max /EDV^2^		5.29 ± 0.85	7.83 ± 2.13 #	3.02 ± 1.02 #
dPdt /EDV	(mm Hg/s/mL)	30.0 ± 3.0	62.3 ± 16.1 #	15.3 ± 5.7 #
*VCBO measurement*				
Ees	(mm Hg/mL)	1.04 ± 0.20	1.74 ± 0.54	1.06 ± 0.31
PRSW	(mm Hg)	74.1 ± 10.2	104.0 ± 14.8 #	51.3 ± 12.9 #

Data are presented as mean ± 95% confidence intervals, n = 13. # = p < 0.05 using paired T-test vs. Control. *HR* heart rate, *SV* stroke volume, *CO*, cardiac output; *Ves*, end-systolic volume; *Ved*, end-diastolic volume; *Pes*, left ventricular end-systolic pressure; *Ped*, left ventricular end-diastolic pressure; *dPdt*_*max*_, maximal rate of pressure increase; *EF*, ejection fraction; *SW*, stroke work; *Tau*, isovolumic relaxation time constant; *PHT*, pressure half time during diastole: *dPdt*_*min*_, maximal rate of pressure decrease; *PWR*_*max*_, maximal power; *Ees*, end-systolic elastance; *PRSW*, preload recruitable stroke work.

**Figure 3:**
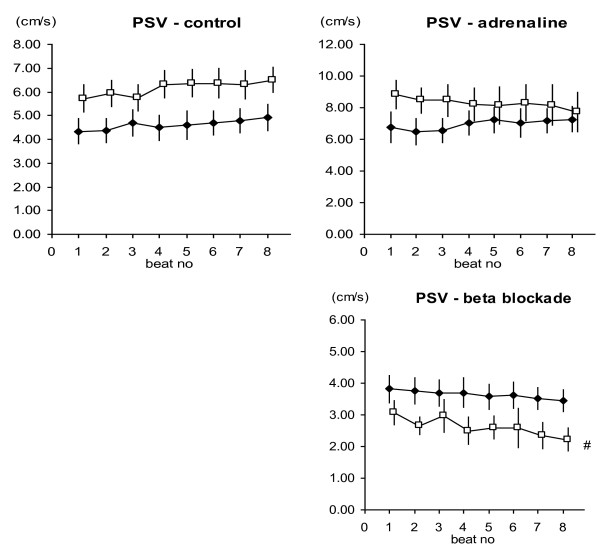
**Peak systolic velocity (PSV) showed no change in PSV value during progressive load reduction for 5 of these 6 groups, and the decrease in PSV for the longitudinal axis measurement in the negative inotropy group was small.** Data are presented as mean ± SEM, n = 13. Filled diamond = Radial projection; Open square = Longitudinal projection. # p < 0.05 with Repeated Measures ANOVA.

The PSV results during the unloading sequence of 8 consecutive beats remained relatively unchanged in both short and long axis at baseline (Figure 3), and behaved in the same manner in the pharmacologically-induced positive inotropic condition. In negative inotropic state, in the short axis results, no significant change in PSV was observed, though in the long axis there was a significant decrease in PSV during the course of the preload reduction sequence.

**Figure 4:**
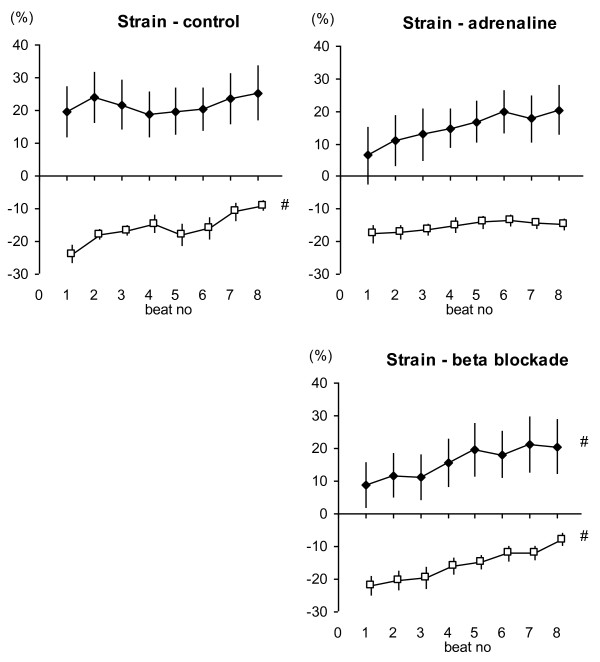
**Systolic Strain.** Systolic strain increased during load reduction in half the group, and notably for the negative inotropy groups. Data are presented as mean ± SEM, n = 13. Filled diamond = Radial projection; Open square = Longitudinal projection. # p < 0.05 with Repeated Measures ANOVA.

Systolic strain findings (Figure 4) showed that during the unloading sequence of 8 consecutive beats, peak systolic strain had a tendency for increase in short axis in baseline and positive inotropic condition, but reached significance only in negative inotropic condition. For the long axis results, a reduction in systolic strain was observed which reached statistical significance in baseline and negative inotropic conditions.

The inotropic interventions led to differences in the tissue\ velocity parameters (Table 2), where PSV increased with the positive inotropic intervention, for both the longitudinal and radial axis measurements. PSV decreased, at least for the radial measurement, with the negative inotropic intervention. There was no clear relation observed for systolic strain and inotropic status. There was no difference between the absolute strain levels for the negative and positive inotropic interventions (Figure 4).

**Table 2:** Effect of inotropic interventions, resting apneic heart cycle

		**Radial projectjon**	**Longitudinal projection**
Control	cm/sec	4.33 ± 1.29	5.72 ± 1.36
Adrenaline	cm/sec	6.77 ± 2.34	8.86 ± 2.18
Beta blockade	cm/sec	3.82 ± 1/02	3.08 ± 0 92

When the radial and longitudinal axes for PSV were compared, there were no differences between the longitudinal axis and radial axis measurements for the resting control and negative inotropic measurements. For the positive inotropic measurement, the PSV average for the longitudinal axis measurement was greater than for the radial axis measurement.

## Discussion

The main findings in this study were that PSV was load-independent in this model. The tissue velocity parameter PSV increased with inotropic status increase, and decreased with the negative inotropic intervention. It has been recognized for many decades that there is a strong relation between load and ventricular performance, though it is not yet clear how strongly related tissue velocities are on ventricular performance. These results support the idea that PSV changes, increases or decreases, reflect changes in contractile status (for example as brought about by inotropic interventions in this model).

Previous reports have been mixed concerning the relation of tissue velocity to load: some pulsed Doppler velocities in the AV plane during load alteration results have supported the idea that PSV is load-independent [16,25-27], others that PSV is load-dependent [28]. Some have performed these studies using human subjects, where although a load or inotropic intervention can be implemented, the actual load-altering effect of these interventions can be difficult to assess and verify [29]. In this experimental model, both loading conditions and contractile status were carefully controlled, and altered independently for serial comparisons.

The results for strain in relation to load supported the idea that strain is load-dependent, in contrast to PSV. Though not reaching statistical significance for all the inotropic conditions, strain appeared to increase generally with decreasing load, in this model, and this agrees with some previously published results [25,30]. There was relatively more variation in the grouped measures for strain (grouped by beat in the preload alteration sequence) compared to PSV. There was relatively less variability in the strain assessments in the longitudinal axis compared to that from the radial axis, and this was expected since conducting measurements in the same tissue plane with the radial axis is more challenging. Strain did not seem to be as affected by inotropic state compared to PSV, also supporting the idea that strain is not a good method for identifying changes in contractile function [31,32].

**Figure 5:**
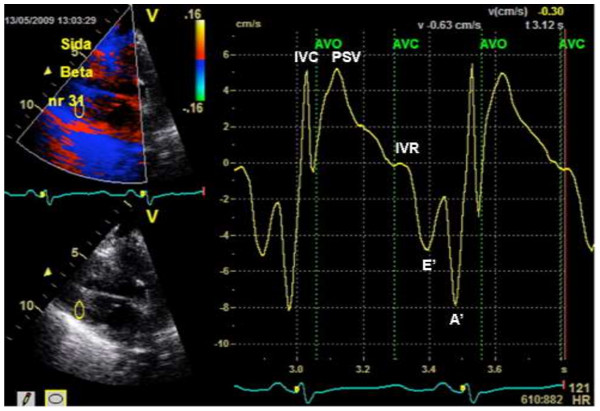
**Tissue velocities, radial axis.** This representative example shows the tissue velocities from the inferior/posterior left ventricular base, with aortic valve opening (AVO) and aortic valve closure (AVC) marked. Isovolumic contraction (IVC) and the peak systolic velocity (PSV) are also shown. Isovolumic relaxation (IVR) is sometimes a subtle finding, and early diastolic velocity (E’) and late diastolic velocity (A’) are marked. E’ was often difficult to identify during the whole preload reduction sequence, as load decreased. **b**. Tissue velocities from apical 4 chamber image, septal base region, signal from multiple beats during vena cava occlusion and adrenaline intervention. PSV coincides with the maximal velocities, which remain relative constant throughout the load reduction (see also progressively changing E’ and A’, which move to a fusion curve during the last beats in the sequence. The goal in signal acquisition (septum) was to be as close to the septal annulus as possible, though as illustrated here, occasionally, signal quality dictated that interrogation was performed a small distance from the annulus. This was accepted since the main findings involve relative changes from beat-to-beat over the load reduction sequence. **c**. Strain in the apical 4 chamber view. The values at zero represent zero deformation during diastole, and the peak systolic strain starts at values of approximately −45% (long axis shortening). In the radial axis, peak systolic strain is positive, starting from a diastolic zero deformation, since the ventricular wall thickens.

There are measurement-technical aspects which can affect the quality of the signals. We analyzed velocities which were measured in the basal aspect of the interventricular septum. Signal from the ventricular lateral wall was also acquired, but there was often inadequate imaging quality during the VCBO, so that we could only consistently use the septal assessment for futher analysis. It is possible that there was some minimal drift of the strain curves over time, since the (Echopac) proprietary software uses drift compensation. We examined signals both with and without drift compensation. Without this software-driven compensation the baseline was consistently drifting. We are confident that the absolute amount of strain was adequately assessed. Strain rate was examined for all VCBO sequences, thought the signal had enough ‘noise’ that clear maximum strain rates were not reliably identified. Therefore, no strain rate results are shown (Figure 5).

This study was conducted with an undisturbed thorax. In cases where the thorax and pericardium are open, it is likely that there is an effect on wall motion and tissue velocities [33]. There have been reports that suggest that transthoracic echocardiographic studies are limited in pigs [31], but imaging was definitely adequate in our experiments with possibly a more modern generation of hardware and software. Also, in a healthy heart, the axis of shortening is not only in the long or short axis [34,35]. It is reasonable perhaps measure in one particular area and then extrapolate to other regions of the heart. In a diseased or injured heart, inhomogeneity of tissue velocities may be known or presumed. In our model, there was not injury. Still, this is not readily generalizable to the clinical setting.

The clinical relevance [35] of these findings is that PSV shows promise as a potential bedside means to obtain a quantitative assessment of contractile function during serial measures in a patient. A recent expert group [36] has suggested that pre-systolic myocardial loading must be taken in account for any clinical application of these parameters. There is a well-recognized degradation of myocardial function, for example with age or illness, which can be followed. Alterations up and down in the range of potential contractile status or function for any individual are part of normal myocardial function, and these can make single beat assessment of general myocardial condition very difficult, since despite implementing an alteration in load, it is much more difficult to absolutely control contractile status, as long as the autonomic nerve system is responsive. On tradition has been to measure single beat parameters in patients at rest, but this may not be adequate for assuring repeatable autonomic nerve system conditions for serial measures. Contractile status needs to be quantitatively assessed, taking advantage of situations where the effect of beat-to-beat loading changes on mechanical systolic function can be approximated. It is possible to do this with high precision measurement of load and mechanical effect for families of beats where load is slightly different for each beat (as has been done here), though the current methodologies for direct ventricular pressure and volume measurements are highly invasive.

This type of assessment is needed today in settings where myocardial dysfunction is suspected, or where inotropic drug intervention is contemplated. Peak systolic velocity shows promise as a relatively load-independent parameter, which reflects systolic mechanical myocardial function, and more clinical experience needs to be gathered.

In summary, these results showed that peak systolic velocities showed a strong load-independence during acute load reduction, though this was not so for strain. We conclude that PSV is a clinically robust parameter of LV regional and global performance under changing load. Peak systolic strain seems to be load-dependent, and has no clear relation to inotropic changes in serial measures. These findings support a broader use of PSV in serial measures in patients to assess changes in ventricular function.

## Competing interest

All authors declare that they have no competing interests concerning this study.

## Authors’ contribution

All authors participated in the initiation and design of the study. All authors execpt JP paricipated in the experiments and data collection. All authors participated in the analysis of the results. All authors contributed to the manuscript, as well as read and approved the final manuscript.
